# Aging does not affect soluble guanylate cyclase redox state in mouse aortas

**DOI:** 10.14814/phy2.12816

**Published:** 2016-05-27

**Authors:** Takashi Shimosato, Masashi Tawa, Hirotaka Iwasaki, Takeshi Imamura, Tomio Okamura

**Affiliations:** ^1^ Department of PharmacologyShiga University of Medical ScienceOtsuShigaJapan

**Keywords:** Aging, nitric oxide, sGC activator, sGC stimulator, soluble guanylate cyclase

## Abstract

Aging is associated with endothelial dysfunction, defined as a reduction in nitric oxide (NO) bioavailability. Although the redox state of the NO acceptor soluble guanylate cyclase (sGC) is another determinant factor for its bioavailability and is disturbed by reactive oxygen species (ROS) known to be increased with age, it is unclear whether aging actually has an impact on vascular sGC redox equilibrium. Therefore, this study investigated this issue using two different types of compounds, the sGC stimulator BAY 41‐2272 and the sGC activator BAY 60‐2770. Plasma thiobarbituric acid‐reactive substances (TBARS) levels were markedly higher in aged (19–20 months old) mice than in young (2–3 months old) mice, whereas superoxide levels in endothelium‐denuded aortas were not different between the groups. The relaxant response of endothelium‐denuded aortas to either BAY 41‐2272 or BAY 60‐2770 was identical in aged and young mice. In addition, the vascular cGMP production stimulated with BAY 41‐2272 or BAY 60‐2770 in aged mice was the same level as that in young mice. These findings suggest that aging accompanied by an increase in systemic oxidative stress does not affect vascular smooth muscle ROS generation and sGC redox equilibrium. Unless ROS are increased in vascular smooth muscle, the sGC redox equilibrium might remain unchanged.

## Introduction

Aging is closely linked to a number of cardiovascular diseases, including atherosclerosis, hypertension, and stroke (Lakatta and Levy 2003; Wang and Bennett 2012). A prominent feature of aging‐induced vascular dysfunction is the reduced bioavailability of nitric oxide (NO), an important endothelium‐derived mediator, and several underlying mechanisms have been proposed. The best‐known one is the prevention of its production. NO is predominantly generated by endothelial NO synthase (eNOS), and aging has been demonstrated to lead to vascular eNOS dysfunction through multiple mechanisms (Soucy et al. 2006; Yang et al. 2009; Lesniewski et al. 2011; Shin et al. 2012). NO disruption is another typical mechanism influencing the effect of this gaseous messenger. It has been reported that superoxide, the primary and most abundant reactive oxygen species (ROS), is increased by aging, resulting in NO scavenging (Francia et al. 2004; Donato et al. 2013).

NO exerts its physiological function mainly through the activation of soluble guanylate cyclase (sGC) and the subsequent cGMP production (Moncada and Higgs 2006). However, a previous study shows that under a certain stress condition like elevated levels of superoxide, the heme moiety of sGC changes from NO‐sensitive to ‐insensitive form (Tawa et al. 2015a). Briefly, there are three configurations: reduced form, which contains a heme moiety with a ferrous iron (Fe^2+^); oxidized form, which contains a heme moiety with a ferric iron (Fe^3+^); and heme‐free form, which does not contain a heme group (Evgenov et al. 2006). Since NO cannot bind to or activate the latter forms, a shift of sGC from the reduced (NO‐sensitive) state to the oxidized/heme‐free (NO‐insensitive) state has attracted attention recently as a determinant of NO bioavailability. In fact, this phenomenon has been confirmed in atherosclerotic, hypertensive, and stroke animal models (Stasch et al. 2006; Casas et al. 2015). On the other hand, it remains unclear whether healthy aging disrupts the sGC redox state, namely the balance between NO‐sensitive and ‐insensitive forms, thus its elucidation is of great interest (Mikhed et al. 2015).

The determinant in the sGC activation by NO is not its heme redox state only (Fernhoff et al. 2009). In other words, it is not always true that the sGC redox equilibrium is normal even if the response to NO is unaltered. This means that NO‐independent sGC stimulants are necessary to evaluate the redox state of sGC. This study, therefore, investigated the influence of aging on vascular sGC redox homeostasis using the reduced sGC stimulant BAY 41‐2272 (Stasch et al. 2001) and the oxidized/heme‐free sGC stimulant BAY 60‐2770 (Knorr et al. 2008). Of course, either the former or the latter can exert its effect in an NO‐independent manner (Evgenov et al. 2006).

## Materials and Methods

### Animals

A total of 48 male ddY mice (1–2 months old) were purchased from Japan SLC, Inc. (Shizuoka, Japan) and were kept at the Research Center for Animal Life Science, Shiga University of Medical Science until experiments were performed. Young and aged animals were used for the experiments at 2–3 months and 19–20 months of age, respectively. The animals were housed in a light‐controlled specific pathogen‐free room with a 12‐h light/dark cycle and were allowed ad libitum access to food (CE‐2, CLEA Japan, Inc., Tokyo, Japan) and water. The Animal Care and Use Committee at Shiga University of Medical Science approved the use of mouse materials along with the experimental protocols in this study (Permit Number: 2014‐4‐9).

### Sample collection

Animals were deeply anesthetized with sodium pentobarbital (50 mg/kg, i.p.) and blood samples were collected from the inferior vena cava. They were then killed by bleeding from the carotid artery. Thoracic aortas were dissected free, excised, and cut helically into strips. The endothelium was removed by gently rubbing the intimal surface with a cotton ball in order to exclude the influence of endothelium‐derived NO. This is because the presence of NO makes it difficult to assess the sGC redox state using BAY compounds (Tawa et al. 2015b).

### Plasma biochemical profile

Blood samples were mixed with heparin and centrifuged at 1500 *g* for 10 min at 4°C. Plasma glucose, total cholesterol, triglycerides, and free fatty acids were measured by SRL Inc. (Tokyo, Japan).

### Assessment of ROS formation

Plasma lipid peroxide levels were estimated using a commercial thiobarbituric acid reactive substances (TBARS) assay kit (Cayman Chemical Co., Ann Arbor, MI). Briefly, the plasma samples were incubated for 1 h at 95°C with thiobarbituric acid under acidic conditions. After cooling and centrifugation at 1500 *g* for 10 min, the malondialdehyde (MDA)‐TBA adduct was measured colorimetrically at 532 nm. TBA reactivity was calculated as micromole MDA per litter.

Superoxide production in aortic segments was determined by measuring lucigenin‐enhanced chemiluminescence (LEC) with a luminometer (Plate CHAMELEON^™^, Hidex, Finland). Segments of thoracic aorta were placed in wells of a white 24‐well plate (24 OptiPlate, Hidex) containing modified Krebs/HEPES solution saturated with 95%O_2_/5%CO_2_, pH 7.4 at 37°C. After a 15‐min equilibration period in the dark, lucigenin (50 μmol/L) was added by auto‐injection into each well and the output of the LEC was measured. The measurements for each well were expressed as counts per second (CPS). Background counts were determined by tissue‐free incubations and subtracted from the readings obtained using tissue. The tissue was dried at 50°C for 24 h and then the dry weight was measured. Chemiluminescence counts were expressed as CPS per mg of dry weight of tissue.

### Vascular reactivity

Aortic strips were fixed vertically between hooks in a muscle bath (10‐mL capacity) containing modified Ringer‐Locke solution with the following composition (mmol/L): NaCl 120, KCl 5.4, CaCl_2_ 2.2, MgCl_2_ 1.0, NaHCO_3_ 25.0, and glucose 5.6. The solution was bubbled with a gas mixture of 95% O_2_ and 5% CO_2_ (pH 7.4), and the temperature was maintained at 37 ± 0.3°C. The hook anchoring the upper end of the strip was connected to the lever of a force‐displacement transducer (Nihon Kohden Kogyo Co., Tokyo, Japan). The resting tension was adjusted to 1.0 g, which is optimal for inducing the maximal contraction. Endothelial denudation was confirmed by the lack of acetylcholine (1 μmol/L)‐induced relaxation of arterial strips that contracted with KCl (30 mmol/L). Before starting the experiments, all the preparations were allowed to equilibrate in the bathing medium for 60–90 min, during which time the solution was replaced every 10–15 min.

The strips were partially contracted with phenylephrine. After the contraction reached a plateau, concentration–response curves for BAY 41‐2272 (sGC stimulator) and BAY 60‐2770 (sGC activator) were obtained by adding the drug directly to the bathing media in cumulative concentrations. At the end of each experiment, papaverine (100 μmol/L) was added to induce the maximal relaxation, which was taken as 100% for relaxations induced by the agonists.

### Measurement of cGMP level

Aortic strips were incubated without (referred to as “basal”) and with BAY 41‐2272 (10^−7^ mol/L) or BAY 60‐2770 (10^−9^ mol/L) for about 20 min according to the above‐described method and were then immediately plunged into liquid nitrogen. The tissues were homogenized in 0.3 mL of 5% trichloroacetic acid at 0°C with a glass homogenizer. After centrifugation at 1500 *g* for 10 min, the supernatant was extracted with water‐saturated ether. The residual ether was removed from the aqueous layer by heating the supernatant for 5 min to 70°C. An aliquot of the extract was then used for determination of cGMP, using a commercial enzyme immunoassay kit (Cayman Chemical Co.). The cGMP level in the tissue was expressed as the relative value divided by the protein content measured in the same extract.

### Pharmacological compounds

The following drugs were used: acetylcholine (Daiichi‐Sankyo Co., Tokyo, Japan); phenylephrine and lucigenin (Sigma Chemical Co., St. Louis, MO); BAY 41‐2272 and BAY 60‐2770 (kindly provided by Dr. Johannes‐Peter Stasch of the Institute of Cardiovascular Research, Pharma Research Centre, Bayer AG, Wuppertal, Germany); papaverine hydrochloride (Dainippon‐Sumitomo Pharma Co., Osaka, Japan); sodium pentobarbital (Kyoritsu Seiyaku Co., Tokyo, Japan); heparin (Mitsubishi Tanabe Pharma Co., Osaka, Japan). Dimethyl sulfoxide was used as a solvent for BAY compounds. Distilled water was used to dissolve all other drugs and to prepare serial dilutions, as required, from stocks on the day of the experiment.

### Statistics

All values are expressed as the mean ± SEM. Concentration–response curves were analyzed by nonlinear curve fitting using Graph Pad Prism 6.0 software (Graph Pad Software Inc., San Diego, CA). The maximal response (*E*_max_) and the negative logarithm of the dilator concentration that caused half of the maximal response (pD_2_) were obtained. Concentration–response curves were analyzed using two‐way repeated measures analysis of variance (ANOVA) and Bonferroni post hoc test. Comparisons between two groups were performed with an unpaired two‐tailed Student's *t*‐test. Differences were considered significant at *P* < 0.05.

## Results

### Influence of aging on metabolic parameters

Body weight was substantially greater in aged mice than in young mice (Table 1). Fasting glucose levels were comparable between the groups, though triglycerides, free fatty acids, and total cholesterol levels were significantly or nearly significantly lower in aged mice (Table 1).

**Table 1:** Metabolic parameters

	Young	Aged	*P* value
Body weight (g)	41.2 ± 0.9 (*n* = 24)	48.7 ± 1.2 (*n* = 24)	<0.001
Glucose (mg/dL)	146.0 ± 7.0 (*n* = 6)	143.5 ± 8.1 (*n* = 6)	0.820
Triglycerides (mg/dL)	149.3 ± 36.7 (*n* = 6)	40.0 ± 9.2 (*n* = 6)	0.016
Free fatty acids (*μ*EQ/L)	741.5 ± 79.5 (*n* = 6)	483.8 ± 56.3 (*n* = 6)	0.025
Total cholesterol (mg/dL)	138.7 ± 9.7 (*n* = 6)	102.0 ± 14.7 (*n* = 6)	0.064

Data are the mean ± SEM.

### Influence of aging on ROS formation

**Figure 1:**
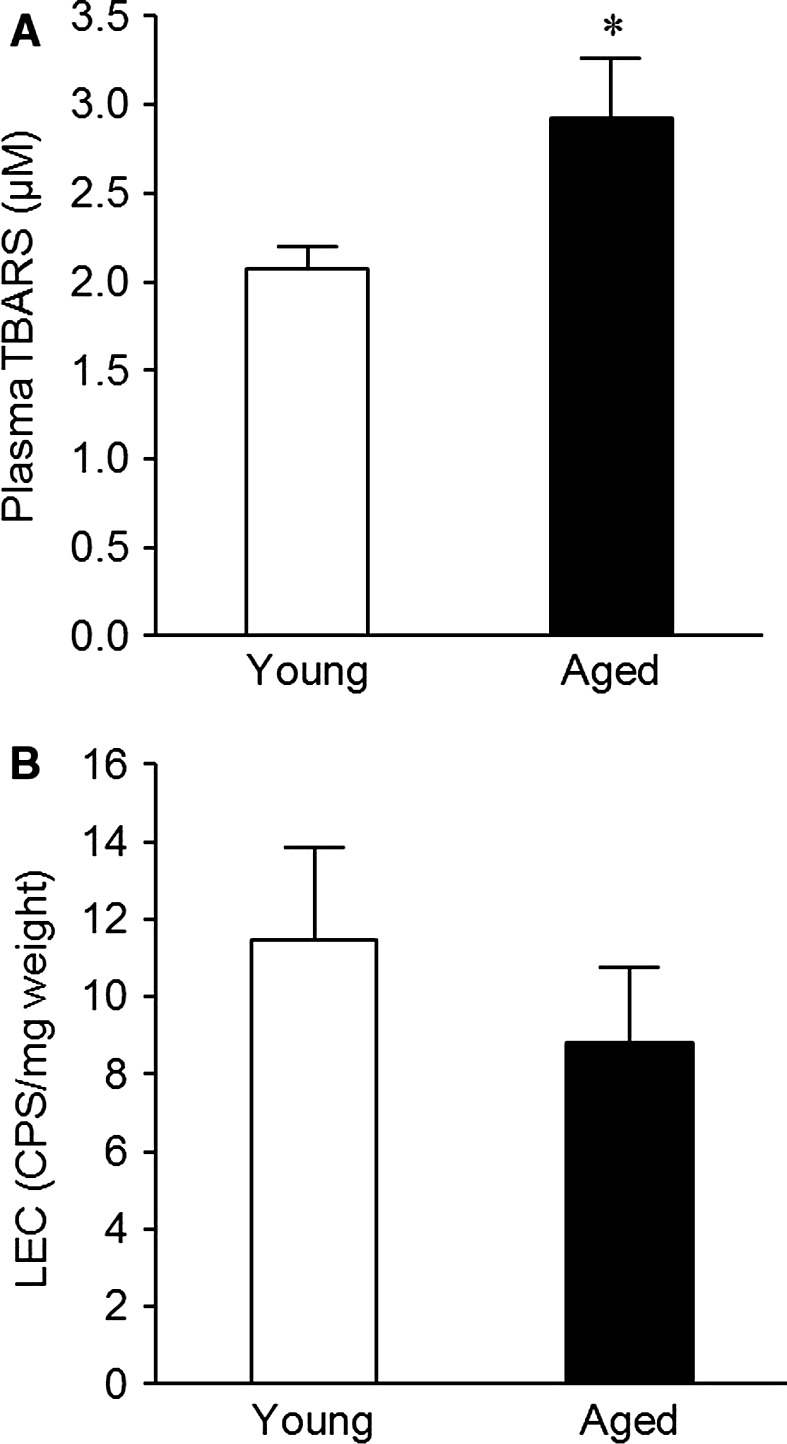
Plasma TBARS levels (A) and endothelium‐denuded aortic superoxide production (B) in young (white column) and aged (black column) mice. Each column and bar represents the mean ± SEM of six experiments. *P < 0.05, compared with the young group.

As shown in Figure 1A, plasma TBARS levels, a marker of systemic oxidative stress, were markedly higher in aged mice than in young mice. On the other hand, Figure 1B shows that a local increase in smooth muscle superoxide production was not observed in aorta from aged mice.

### Influence of aging on vascular reactivity

**Figure 2:**
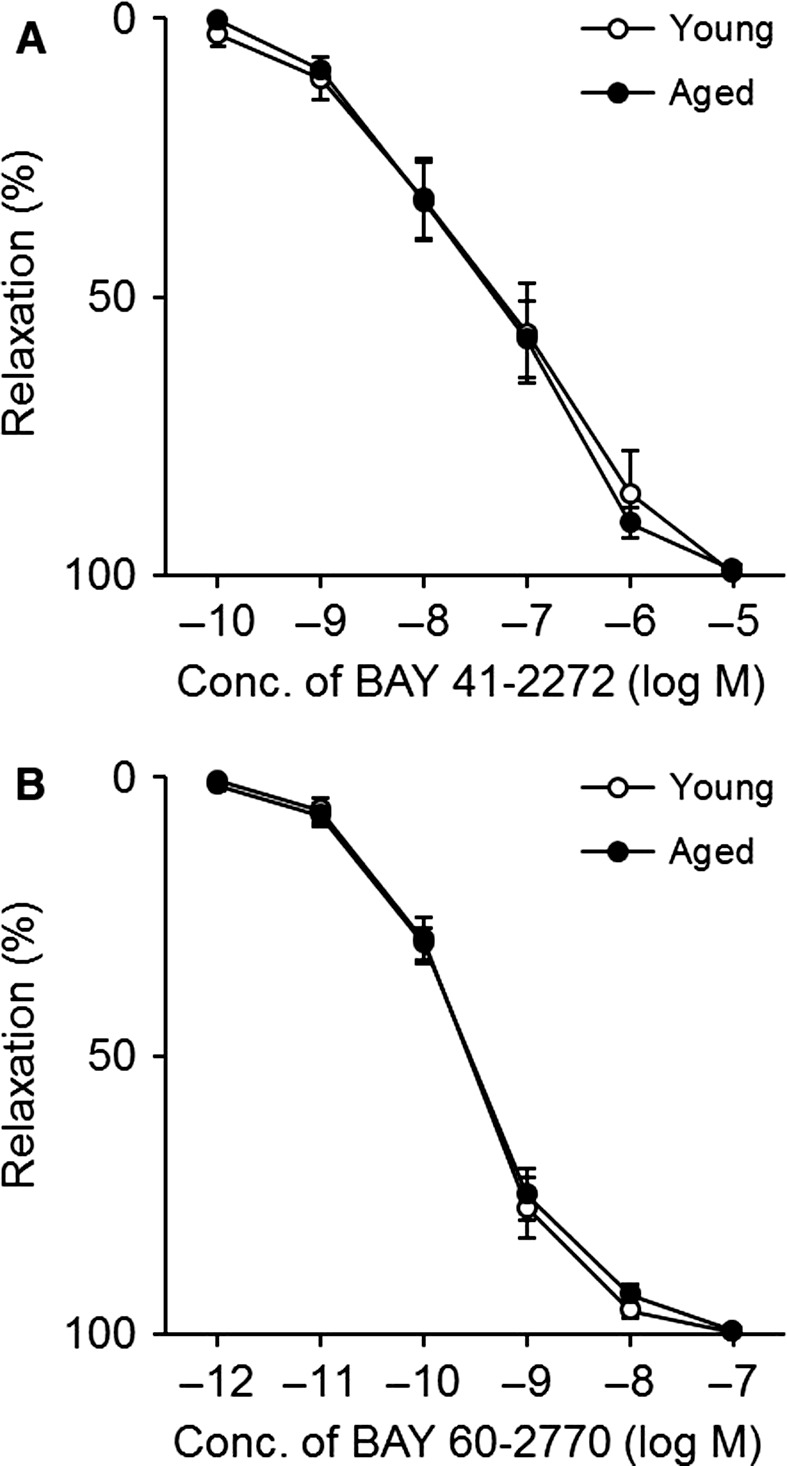
Concentration–response curves to BAY 41‐2272 (A) and BAY 60‐2770 (B) in young (white circle) and aged (black circle) endothelium‐denuded mouse aortas. Each point and bar represents the mean ± SEM of six experiments.

Either BAY 41‐2272 or BAY 60‐2770 produced a concentration‐dependent relaxation of endothelium‐denuded aortas, which was not different between young and aged mice (Fig. 2A and B). The efficacy and potency of BAY compounds were also identical between the groups (Table 2).

**Table 2:** pD_2_ and *E*_max_ to relaxant agonists

	Young	Aged	*P* value
BAY 41‐2272			
pD_2_	7.18 ± 0.32 (*n* = 6)	7.38 ± 0.24 (*n* = 6)	0.632
*E*_max_ (%)	99.8 ± 2.8 (*n* = 6)	96.4 ± 1.8 (*n* = 6)	0.340
BAY 60‐2770			
pD_2_	9.57 ± 0.11 (*n* = 6)	9.55 ± 0.08 (*n* = 6)	0.843
*E*_max_ (%)	99.3 ± 1.0 (*n* = 6)	97.5 ± 0.9 (*n* = 6)	0.222

Data are the mean ± SEM.

### Influence of aging on cGMP production

**Figure 3:**
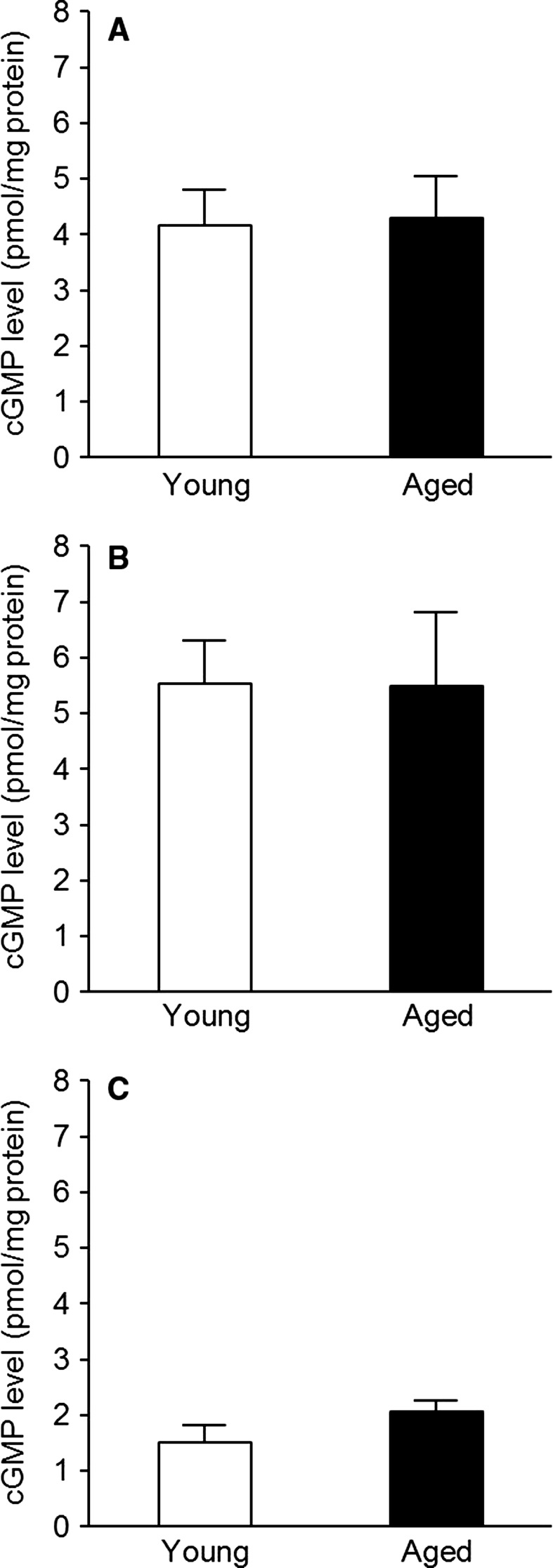
cGMP levels after stimulation with BAY 41‐2272 (A), BAY 60‐2770 (B), and no drug (C) in young (white column) and aged (black column) endothelium‐denuded mouse aortas. Each column and bar represents the mean ± SEM of six experiments.

cGMP levels in endothelium‐denuded aortas stimulated with 10^−7^ mol/L BAY 41‐2272 (Fig. 3A) or 10^−9^ mol/L BAY 60‐2770 (Fig. 3B) in aged mice were not different from those in young mice. By the way, there was also no significant difference in basal levels of cGMP among young and aged groups (Fig. 3C).

## Discussion

The main findings of this study are that (1) aging is accompanied by an increase in plasma TBARS levels in mice, (2) aging does not induce superoxide overproduction in mouse aortas without endothelium, and (3) aging has no influence on vascular responses of endothelium‐denuded mouse aortas to BAY 41‐2272 and BAY 60‐2770.

A key signal transduction enzyme sGC can exist in the reduced, oxidized, and heme‐free forms (Evgenov et al. 2006), yet there is no way at this time to quantify the relative expression ratio of these three types. Because NO is now recognized to serve as a ligand for sGC but cannot activate the oxidized/heme‐free form (Evgenov et al. 2006), it is very important to figure out its balance. To overcome this issue, sGC stimulators and sGC activators are used widely as tools for evaluating the redox state of sGC (Evgenov et al. 2006; Tawa et al. 2014; Tawa et al. 2015a,b), and these two types of drugs were also utilized in this study. As a result, relaxation and cGMP formation of endothelium‐denuded aortas in response to either the sGC stimulator BAY 41‐2272 or the sGC activator BAY 60‐2770 were comparable in young and aged mice, suggesting that vascular sGC redox equilibrium is not affected by aging. To the best of our knowledge, there is no report investigating the effects of an sGC stimulator or an sGC activator in blood vessels of healthy aged individuals. Nevertheless, the above conclusion is supported by the finding that vascular smooth muscle response to NO is unaffected by aging (Stewart et al. 2000; Ferrer and Balfagón 2001; Reyes‐Toso et al. 2007). If the redox state of sGC is disrupted, NO‐induced effects are supposed to be decreased.

A certain type of ROS has been proved to shift vascular sGC from the NO‐sensitive state to the NO‐insensitive state (Stasch et al. 2006; Tawa et al. 2014; Tawa et al. 2015a,b). As widely known, aging is closely associated with increased ROS production. This study confirmed that circulating TBARS levels in aged mice are significantly higher than those in young mice, being in line with previous reports (Poubelle et al. 1982; Yamato et al. 2014). However, it is not always true that ROS is produced everywhere. Actually, aging did not induce superoxide overproduction in endothelium‐denuded mouse aortas, and the same result has been found in many different studies (van der Loo et al. 2000; Mukai et al. 2002), suggesting that the smooth muscle is not a primary site of age‐related ROS generation. In regard to this point, we have demonstrated that if superoxide is not increased intracellularly in vascular smooth muscle, a shift of the sGC redox state does not happen (Tawa et al. 2015a). Altogether, a parameter that we should pay attention to a relationship with sGC might be local, but not systemic, ROS levels. Therefore, it is rational that aging without vascular smooth muscle ROS overproduction has no impact on the redox state of sGC in the vasculature.

The metabolic system, in addition to the circulatory system, is also affected by aging. Indeed, aged mice showed decreased fasting plasma triglycerides, free fatty acids, and total cholesterol levels, whereas the animals had unaltered fasting plasma glucose levels. Consistent with our findings, Stämpfli and colleagues have reported that aging induces a decline in plasma triglycerides and total cholesterol but not blood glucose in mice (Stämpfli et al. 2010). These conditions are not coincident with those observed in general metabolic disorders like hyperlipidemia and diabetes, indicating that aging and such diseases have different pathophysiological feature. Thus, the impact of aging on vascular system might be not exactly the same as that of metabolic disorders. As a matter of fact, diabetes has been demonstrated to disrupt the sGC redox equilibrium in the vasculature (Stasch et al. 2006; Goulopoulou et al. 2015), which is contrasted with the present findings about aging. Needless to say, it is important to understand the respective clinical condition separately.

One limitation of this study is the lack of data in endothelium‐intact preparations. The reason for using endothelium‐denuded, but not endothelium‐intact, mouse aortas in this study is because sGC stimulators and sGC activators produce synergistic and additive effects, respectively, with NO (Evgenov et al. 2006). Briefly, when NO is continuously released from the endothelial cells, that will get in the way of evaluating the sGC redox state by these drugs. It is true of course that there are reports showing different outcomes between endothelium‐intact and ‐denuded preparations (Tabernero and Vila 1995; Shipley and Muller‐Delp 2005). However, this is thought to be not the case in this study, because the relaxant potency and efficacy of the NO donor sodium nitroprusside in endothelium‐intact mouse aortas were not attenuated by aging (data not shown). That is to say, at least the NO‐sensitive reduced sGC is highly possible to be maintained in aged mouse aortas whether the endothelium is present or absent. Just for information, Reyes‐Toso et al. have also proved that aging does not affect sodium nitroprusside‐induced relaxation of either endothelium‐intact or ‐denuded rat aortas (Reyes‐Toso et al. 2007). In any case, further studies are certainly necessary in order to better understand the influence of aging on vascular sGC redox equilibrium.

In conclusion, this study clearly demonstrates that aged mouse aortic smooth muscle still possesses normal superoxide levels and therefore does not undergo a shift of the sGC redox equilibrium toward the NO‐insensitive state. Although age‐related increase in systemic oxidative stress was found, the phenomenon was shown to be not attributed to disruption of the redox state of sGC in the vasculature.

## Conflict of Interest

None declared.
